# Mortality and exacerbations associated with *Stenotrophomonas maltophilia* in chronic obstructive pulmonary disease. A regional cohort study of 22,689 outpatients

**DOI:** 10.1186/s12931-023-02544-w

**Published:** 2023-09-26

**Authors:** Christian Rønn, Peter Kamstrup, Josefin Eklöf, Louise Lindhardt Toennesen, Jonas Bredtoft Boel, Christian Ostergaard Andersen, Ram Benny Dessau, Jon Torgny Wilcke, Pradeesh Sivapalan, Charlotte Suppli Ulrik, Jens-Ulrik Stæhr Jensen

**Affiliations:** 1 Section of Respiratory Medicine, Department of Medicine, Copenhagen University Hospital – Gentofte, Hellerup, Denmark; 2 Department of Clinical Microbiology, Copenhagen University Hospital - Herlev, Herlev, Denmark; 3 Department of Clinical Microbiology, Copenhagen University Hospital - Hvidovre, Hvidovre, Denmark; 4 Department of Clinical Microbiology, Zealand University Hospital, Slagelse, Denmark; 5 Department of Regional Health Research, University of Southern Denmark, Odense, Denmark; 6 Department of Respiratory Medicine, Copenhagen University Hospital - Hvidovre, Hvidovre, Denmark; 7 Department of Clinical Medicine, Faculty of Health and Medical Sciences, University of Copenhagen, Copenhagen, Denmark

## Abstract

**Objectives:**

The clinical significance of *Stenotrophomonas maltophilia* in patients with COPD is poorly understood. We aimed to determine whether a lower respiratory tract culture positive for *S. maltophilia* in COPD patients was independently associated with increased risk of death and hospitalisation for exacerbation of COPD.

**Methods:**

An observational cohort study following outpatients with COPD in Eastern Denmark between 2010 and 2018, with a follow-up period of five years. Presence of S. maltophilia was treated as a time-varying exposure, where patients were considered exposed at the time of the first isolation of S. maltophilia from the lower respiratory tract. The hazard ratio (HR) of death and hospitalisation for acute exacerbations of COPD was assessed using a Cox proportional hazards regression.

**Results:**

Of the total 22,689 patients 459 (2.0%) had a lower respiratory sample positive for *S. maltophilia*. A total of 7,649 deaths (*S. maltophilia* positive: 243 (52.9%) and *S. maltophilia* negative: 7,406 (34.4%)) and 24,912 hospitalisations for exacerbation of COPD (*S. maltophilia* positive: 1,100 in 459 patients and *S. maltophilia* negative: 23,821 in 22,230 patients) were registered during the study period. We found that a lower respiratory tract culture positive for *S. maltophilia* was associated with both increased mortality: HR 3.3 (95% CI 2.6–4.3), and hospitalisation for exacerbation of COPD: HR 3.4 (95% CI 2.8–4.1).

**Conclusions:**

A lower respiratory tract culture positive for *S. maltophilia* in COPD patients was associated with a substantially increased mortality and hospitalisation for exacerbation of COPD. Randomised controlled trials are proposed to determine whether S. maltophilia should be the target of antibiotic treatment.

**Supplementary Information:**

The online version contains supplementary material available at 10.1186/s12931-023-02544-w.

## Introduction

*Stenotrophomonas maltophilia* is an ubiquitous, aerobic, non-fermentative, Gram-negative bacillus, first discovered in 1943 [1]. *S. maltophilia* is an important nosocomial pathogen associated with crude mortality in the hospitalised ranging between 14 and 69% [2] with an attributable mortality rate of up to 37.5% [3].

The role of *S. maltophilia* in patients with chronic obstructive pulmonary disease (COPD) is poorly understood, and it is debated whether *S. maltophilia* should be considered a marker of pulmonary disease progression or regarded as a pathogen [4]. However, a recent case report suggested *S. maltophilia* may be a driver of exacerbation in a patient with COPD [5].

In a Canadian population of COPD outpatients, *S. maltophilia* was found in 10% of patients, and associated with a 2-fold increase in mortality [6]. However, the sample size was small (n = 397), and the prevalence was notably higher than earlier findings of 0.5% in stable COPD patients [7].

In patients with non-cystic fibrosis bronchiectasis the presence of *S. maltophilia* is associated with more severe bronchiectasis and more pronounced airway inflammation [8], the same pattern which is seen in patients with cystic fibrosis bronchiectasis [9].

A similar, opportunistic bacterium of the same class of Gammaproteobacteria which has been studied in greater detail, is *Pseudomonas aeruginosa*. Our group has previously shown that *Pseudomonas aeruginosa* in patients with COPD was associated with a 3-fold increased risk of acute exacerbations and a highly increased risk of death which is in line with other studies [10] [11, 12]. Likewise, the presence of *P. aeruginosa* was associated with an increased airway inflammation, more frequent exacerbations and death both in patients with non-cystic fibrosis bronchiectasis [13, 14] as well as patients with cystic fibrosis bronchiectasis [4, 15, 16].

The aim of this study was to determine, in a large cohort of outpatients with COPD with complete follow-up on both outcomes, whether acquiring a lower respiratory tract culture with *S. maltophilia* was associated with increased mortality and hospitalisation for exacerbation of COPD.

## Methods

### Data sources

Data were collected from the following databases:

1. The Danish Register of COPD (DrCOPD) is a nationwide register on all COPD patients at all outpatient visits and all hospital-based respiratory outpatient clinics since 2010 [17]. Patients in DrCOPD have been assessed by a pulmonologist and have a spirometry verified diagnosis of COPD. At each outpatient visit a registered nurse performed spirometry, including measurements of forced expiratory volume in one second percent of predicted (FEV_1_, also expressed as percentage of predicted) and obtained information on Medical Research Council dyspnoea score, body mass index (BMI), and tobacco exposure.
2. The Danish National Patient Registry contains data on all hospital contacts since 1995, including diagnoses and length of contact [18]. Data on co-morbidities, together with hospitalizations due to exacerbations during the study period were obtained from this registry.
3. The Danish National Database of Reimbursed Prescriptions is a nationwide register and includes information on the strength, dose, product name, and Anatomical Therapeutic Chemical classification of each prescription on all redeemed prescriptions [19]. Filled, and reimbursed, prescriptions on inhaled corticosteroids and oral corticosteroids were gathered from this database.
4. Microbiological data from the Clinical Microbiology Departments in Eastern Denmark (Region Zealand and Capital Region) consisting of approximately 2.6 million inhabitants. The register contains information on all microbiological samples, including lower respiratory tract cultures, and by that used to identify patients with *S. maltophilia*.

### Study design

This was a retrospective, registry-based cohort study. The study cohort comprised all COPD patients registered with an outpatient clinic visit in eastern Denmark from 1st January 2010 to 31st October 2017 in DrCOPD. Patients not resident in Eastern Denmark were excluded as we had no access to microbiological data on these patients.

We excluded patients with a history of immunodeficiency (International classification of Diseases 10th revision (ICD-10): D80-D84, D89) and malignancy (ICD-10: C00-C97, except C44) within the last five years from cohort entry, except for non-melanoma malignancies of the skin. Also, patients with a lower respiratory tract sample positive for *S. maltophilia* within 365 days of cohort entry were excluded to avoid differential misclassification.

Cohort entry was defined as the date for the patient’s first outpatient clinic visit recorded in DrCOPD and only stable patients without hospital admission on the date were included. Patients with only in-hospital registrations were excluded since these registrations do not contain information on essential patient characteristics.

Sputum samples were not taken routinely on all patients, but rather when relevant symptoms were present such as cough, phlegm, dyspnoea, fever etc.

Patients were followed for five years or until the first of either (1) end of follow-up on 1st July 2018, or (2) death.

### Statistical analyses

Descriptive statistics were performed on the baseline data at the time of study entry. Comparison between baseline data was performed with a Chi-squared test for categorical data and *t* test for continuous data.

The hazard ratio of death following a lower respiratory tract culture with *S. maltophilia* was estimated using a Cox proportional hazards regression. Presence of *S. maltophilia* was treated as a time-varying exposure, where patients were considered exposed from the time of the first isolation of *S. maltophilia* in lower respiratory tract samples and for the remainder of the follow-up time, since we could not validly define the patient as having cleared the bacteria. The model was adjusted for the following confounders: age (continuous [year]), FEV_1_ (continuous [%]), BMI (continuous [kg/m^2^]), sex (male vs. female), smoking status (active vs. former), severe exacerbations (ICD-10: J44.1) 365 days prior to cohort entry (none, 1, ≥ 2), cumulated dose of budesonide-equivalent ICS 365 days prior to cohort entry (none, ≤ 400 µg, 400–800 µg, > 800 µg), cumulated dose of oral corticosteroids 365 days prior to cohort entry (none, ≤ 250 mg prednisolone, > 250 mg prednisolone), and calendar year for entry in DrCOPD. Cut-off for prednisolone was chosen to reflect a history of exacerbations with “low dose” corresponding to one exacerbation and “high dose” corresponding to two or more exacerbations.

Results are presented as hazard ratios (HR) with 95% confidence intervals (CI) and p-values (P). To assess robustness of the methodology, a propensity score weighted Cox proportional hazards regression was performed as a sensitivity analysis using the same confounders for propensity scoring as was used for adjustment in the Cox proportional hazards regression.

Analysis of the association between acute exacerbations of COPD and a lower respiratory tract culture with *S. maltophilia* was performed with recurrent event Cox proportional hazards regression analysis using an Andersen-Gill model [20] with death included as a censoring event, adjusting for the same confounders as in the primary analyses.

As sensitivity analyses we performed both a complete case Cox proportional hazards regression and a propensity scoreweighted Cox proportional hazards regression in addition to a self-controlled case series [21], including only the patients with a lower respiratory tract culture with *S. maltophilia*, they were followed from one year prior to first lower respiratory tract sample positive for *S. maltophilia* and one year after. Results are presented as incidence rate ratios (IRR) with 95% CI.

### Model validation

The proportional hazards assumption was tested by including a test for interaction with time. Additionally, we tested for linearity of the continuous covariates. Continuous covariates that did not meet the criteria for linearity were handled using penalised splines. Missing data were handled by the substantive model for multiple imputation, with 100 imputations with each 20 iterations [22].

Data management and statistical analyses were performed using Statistical Analysis Software 9.4 (SAS Institute, Cary, NC, USA). Multiple imputation, Cox proportional hazards regression, and inverse probability of treatment weighting were completed in R 4.1.3 (R Foundation for Statistical Computing, Vienna, Austria) with the SCCS 1.6, SMCFCS 1.6.1, MITOOLS 2.4, and SURVIVAL 3.3.1 packages respectively.

## Results

A total of 106,560 patients with COPD were identified in DrCOPD. Of these 57,843 had at least one outpatient visit during the study period. Patients from Western Denmark (n = 32,617) were excluded due to no accessible microbiological data. Additionally, patients with a diagnosis of malignant disease (n = 2,312) or immunodeficiency (n = 68) within five years before study entry were excluded, as well as those with a lower respiratory tract sample positive for *S. maltophilia* (n = 89) within 365 days prior potential cohort entry were excluded.

**Fig. 1:**
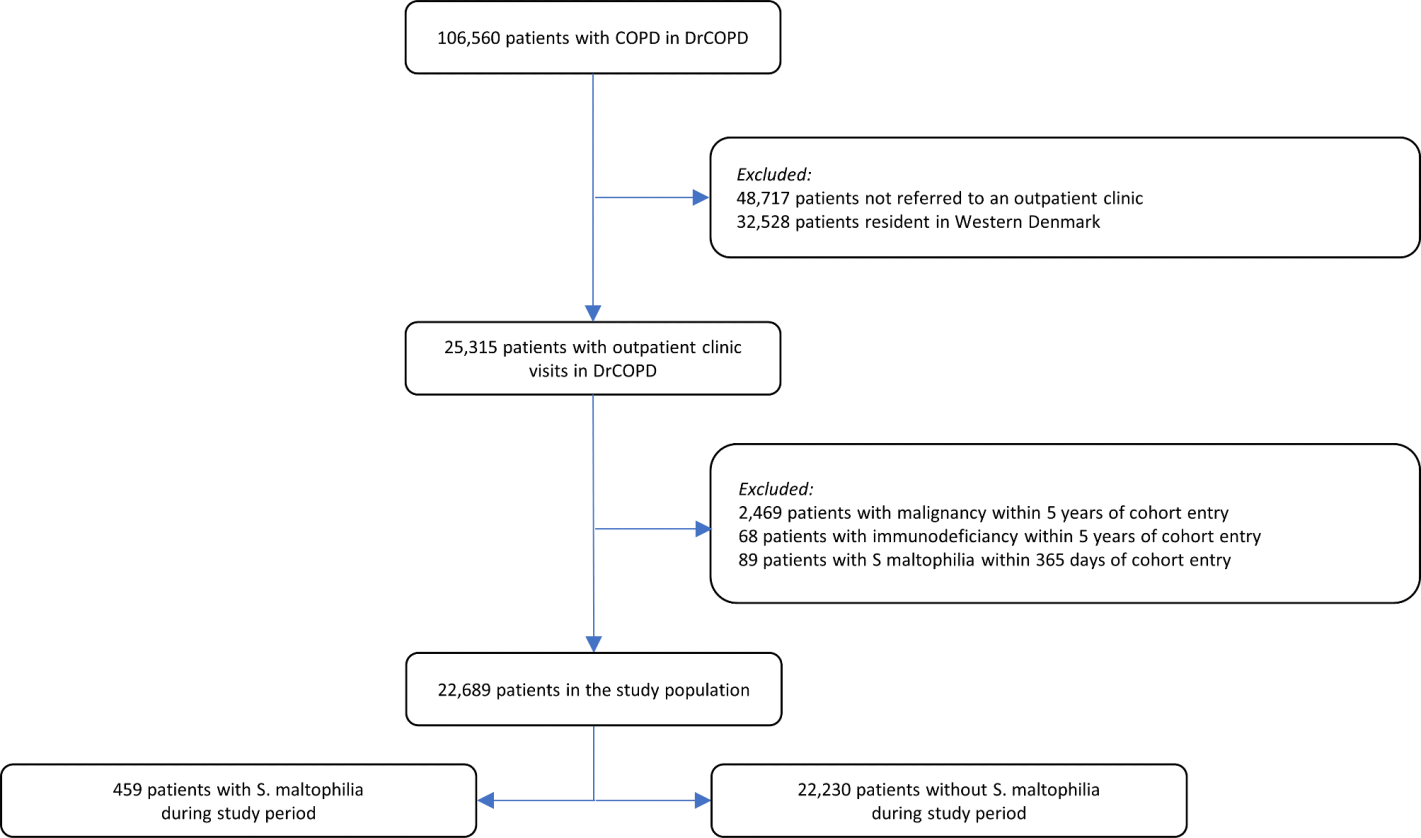
Study flowchart. DrCOPD: Danish register of COPD.

The final study cohort comprised 22,869 patients that contributed with 81,967 person-years of risk time for the analysis. A positive lower respiratory tract sample with *S. maltophilia* was found in 459 (2.0%) patients (Fig. 1), with median time to first isolation being 673 days (interquartile range: 302–1184 days). Of the 459 patients, 179 (39%) patients had a follow-up respiratory tract sample positive for *S. maltophilia*. In the remaining 280 (61%) patients *S. maltophilia* was not found after the initial isolation – 47 (17%) patients had a follow-up respiratory tract sample negative for *S. maltophilia*, while 223 (83%) patients had no recorded follow-up respiratory tract sample. A total of 7,649 deaths (*S. maltophilia* positive: 243 (52.9%) and *S. maltophilia* negative: 7,406 (34.4%)) and 24,912 hospitalisations for exacerbation of COPD (*S. maltophilia* positive: 1,100 in 459 patients and *S. maltophilia* negative: 23,821 in 22,230 patients) were registered during the study period. For baseline characteristics see Table 1, and definitions of comorbidities see Supplemental Appendix 1.

**Table 1:** Baseline characteristics at study entry of COPD outpatients with samples positive or negative for *S. maltophilia*

	No sample/sample negative for *S. maltophilia* (n=22,230)	Sample positive for *S. maltophilia* (n=459)	P-value
Sex female, n (%)	12,108 (54.5)	267 (58.2)	0.116
Age (years), median (IQR)	69.8 (61.9–77.2)	69.6 (64.1–74.7)	0.608
FEV_1_ (%), median (IQR), missing [%]	50 (36–63) [11.6]	37 (28–49) [3.7]	<0.0001
BMI (kg/m^2^), median (IQR) missing [%]	25 (21–29) [12.3]	23 (20–27) [4.4]	<0.0001
MRC, median (IQR)	3 (2–4)	3 (3–4)	<0.0001
Smoking status			<0.0001
Former smoker, n (%)	12,218 (55.0)	313 (68.2)	
Active smoker, n (%)	7,484 (33.7)	122 (26.6)	
Smoking status missing, n (%)	2,525 (11.3)	24 (5.2)	
Severe exacerbations			<0.0001
0, n (%)	14,748 (67.0)	244 (48.8)	
1, n (%)	2,763 (12.5)	63 (13.7)	
≥2, n (%)	4,510 (20.5)	172 (37.5)	
Inhaled corticosteroid⁎			<0.0001
None, n (%)	7,197 (32.7)	38 (8.3)	
Low, n (%)	6,174 (28.0)	95 (20.7)	
Moderate, n (%)	4,665 (21.2)	128 (27.9)	
High, n (%)	3,985 (18,1)	198 (43.1)	
Oral corticosteroid⁑			<0.0001
None, n (%)	14,224 (64.0)	185 (40.3)	
Low dose, n (%)	2,178 (9.8)	42 (9.2)	
High dose, n (%)	5,822 (26.2)	232 (50.5)	
Comorbidities⁂			
Cerebrovascular disease, n (%)	1,623 (7.3)	41 (7.2)	0.900
Asthma, n (%)	3,267 (14.7)	64 (13.9)	0.674
Atrial fibrillation, n (%)	3,207 (14.4)	53 (11.5)	0.088
Depression, n (%)	1,062 (4.8)	17 (3.7)	0.290
Diabetes mellitus, n (%)	2,623 (11.8)	51 (11.1)	0.666
Congestive heart failure, n (%)	3,710 (16.7)	60 (13.1)	0.043
Ischaemic heart disease, n (%)	1,623 (7.3)	33 (7.2)	0.921
Renal disease, n (%)	1,017 (4.6)	19 (4.1)	0.676
Peripheral vascular disease, n (%)	2,220 (10.0)	47 (10.2)	0.845

*S. maltophilia*: *Stenotrophomonas maltophilia*, n: number, IQR: interquartile range, FEV1: forced expired volume in the first second, MRC: Medical Research Council dyspnoea scale, BMI: body mass index, ⁎ cumulated dose of budesonide-equivalent ICS 365 days prior to study entry, none: no use, low: ≤400 µg, moderate: 400–800 µg, high: >800 µg ⁑Oral corticosteroids accumulated dose 365 prior to study entry, none: no use, low dose: ≤ 250 mg prednisolone, high dose: > 250 mg prednisolone ⁂ Comorbidities registered in the Danish National Patient Registry prior to study entry (Supplemental Appendix 1)

**Fig. 2:**
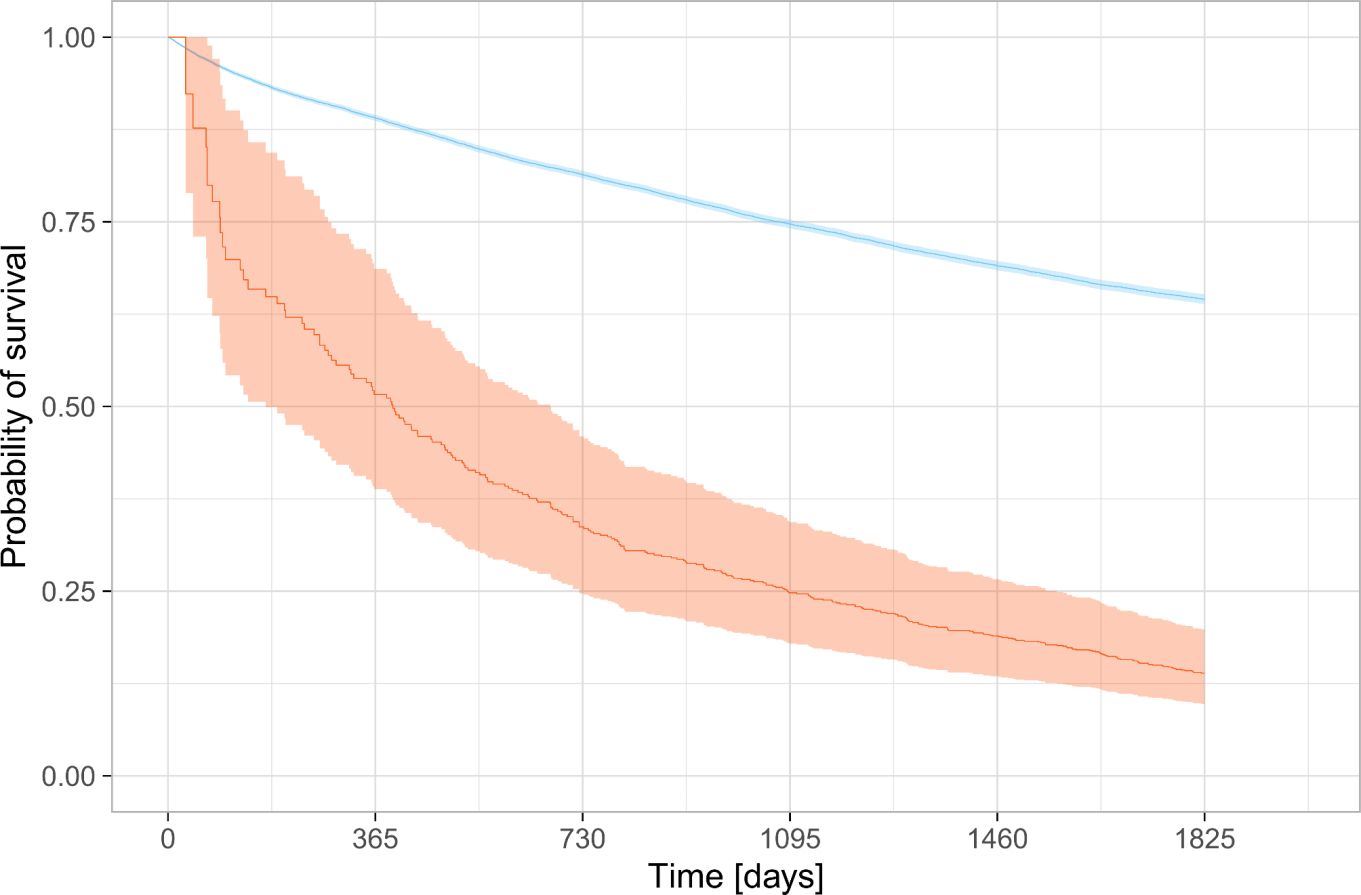
Simon-Makuch plot of *overall survival five years from first outpatient clinic visit* according to *S. maltophilia* status in lower respiratory tract sample (red: sample positive for *S. maltophilia* and blue: no sample/sample not positive for *S. maltophilia*. Mean in solid, 95% confidence interval in transparent)

**Fig. 3:**
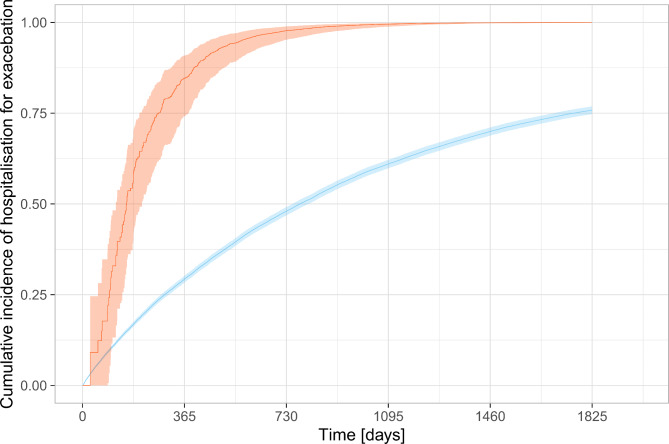
Cumulative incidence of hospitalisation of exacerbation of COPD *five years from first outpatient clinic visit* according to *S. maltophilia* status in lower respiratory tract samples (red: sample positive for *S. maltophilia* and blue: no sample/sample not positive for *S. maltophilia*. Mean in solid, 95% confidence interval in transparent)

 The adjusted Cox proportional hazards regression showed an increased hazard of death: HR 2.6 (95% CI 2.2–3.1, P < 0.0001) and hospitalisation for exacerbation: HR 2.7 (95% CI 2.3–3.1, P < 0.0001) following a positive lower respiratory tract sample for *S. maltophilia* which were an attenuation of the unadjusted results (Table 2; Figs. 2 and 3. For full adjusted analysis Supplemental Appendix 2). The complete case Cox proportional hazards regression did not change the findings.

**Table 2:** Hazard for death or hospitalisation with exacerbation of COPD with acquisition of *S. maltophilia*

	Unadjusted			Adjusted			PS weighted		
	HR	CI	P	HR	CI	P	HR	CI	P
Death	4.2	3.6-4.8	<0.0001	2.6	2.2-3.1	<0.0001	3.7	3.1-4.3	<0.0001
Hospitalisation	5.7	5.0-6.6	<0.0001	2.7	2.3-3.1	<0.0001	3.3	2.9-3.7	<0.0001

*S. maltophilia: Stenotrophomonas maltophilia*, PS: propensity score, HR: hazard ratio, CI: 95% confidence interval, P: p-value. The analysis was adjusted for: age, sex, forced expired volume in the first second, body mass index, smoking status, severe exacerbations, inhaled and oral corticosteroids use, and calendar year for cohort entry

The PS weighted sensitivity analysis confirmed the main finding: HR for death 3.7 (95% CI 3.1–4.3, P < 0.0001) and HR for hospitalisation 3.3(95% CI 2.7–3.7, P < 0.0001).

Additionally, a self-controlled case series analysis was performed as a further sensitivity analysis on the hospitalisation outcome, which slightly attenuated the finding: IRR 1.8, (95% CI 1.4–2.2, P < 0.0001).

Of the included patients, 19,757 (87%) had complete data on all data variables. The remaining 2,932 (13%) had one or more clinical values missing from the outpatient clinic visit. Most patients with missing data had no data on either FEV1, BMI, and smoking status.

## Discussion

In this registry-based cohort study of outpatients with COPD we found that a lower respiratory tract sample positive for *S. maltophilia* was strongly and independently associated with a marked increase in mortality as well as hospitalisation for exacerbation of COPD, both results robust for adjustment and sensitivity analysis.

The evidence on COPD and the role of *S. maltophilia* is sparse. A Canadian study screened all COPD patients on their visits to the respiratory outpatient clinic and found a sputum sample positive for *S. maltophilia* in 10%. *S. maltophilia* was associated with a 2-fold increase in mortality, however, the sample size was small (n = 397) and notably the prevalence of *S. maltophilia* (10%) was markedly higher than earlier findings in stable COPD patients including in the present study (0.5-2.0%) [7]. Also, no other clinical outcomes were reported. Interestingly, even with 15% having a malignant diagnosis, the mortality rate during mean follow-up of 2 years (*S. maltophilia*: 22%, controls: 7%) was notably lower than in our study (*S. maltophilia*: 60%, controls: 20%) while base line data were similar. The two year mortality rate of COPD patients have previously been reported to be 16% in stable COPD patients [23] increasing to 35–55% in COPD patients following hospitalisation for exacerbation [24, 25].

In other chronic pulmonary diseases, *S. maltophilia* in the lower respiratory tract has been associated with deteriorating clinical end points. A recent study investigating the sputum microbiome and clinical outcomes in patients with bronchiectasis showed that *S. maltophilia* was associated with more severe bronchiectasis and a higher degree of lung inflammation [8]. In patients with cystic fibrosis bronchiectasis, *S. maltophilia* is a well-established contributor to irreversible decline in lung function and increase in exacerbations [15, 16]. The pathogenesis is closely linked to well-studied and complex virulence mechanisms, cross-infection, and multi-resistance [4].

Strengths of the current study include observations based on a large and well-characterized population of patients with a respiratory specialist verified and spirometry confirmed diagnosis of COPD. The patients had two or more outpatient clinic visits but were, in all other aspects, unselected, likely reducing the risk of bias. Further, our registries contain data on many important confounders such as smoking status, oral and inhaled corticosteroid use, FEV_1_, and BMI, allowing us to account for all these, and the completeness of these data was high. The exposure, a lower respiratory tract sample positive for *S. maltophilia*, was well established for the entire cohort, as we had access to all microbiological data from Eastern Denmark during the entire study period. This included all samples cultured from admitted patients, outpatients, emergency department visits and primary care. Last, there was a 100% follow-up on death and hospital admission for exacerbation for COPD which in combination with a large sample size (n = 22,869) minimize the risk of false positive results.

Despite the noted strengths, our study has some important limitations that deserve careful considerations. Firstly, we do not have lower respiratory tract samples from all patients. In our outpatient clinics, it is custom, and described in national guidelines, that all COPD patients with symptoms suggesting an airway infection shall be culture sampled. Accordingly, we do not have follow-up lower respiratory tract samples from approximately half the patients positive for *S. maltophilia.* However, most of the patients who did have a follow-up sample available, 79% remained positive for *S maltophilia.* Secondly, we do not hold data on previous non-hospital requiring exacerbations of COPD, but we account for this by controlling for oral corticosteroid use as a proxy for exacerbations.

In conclusion, we present a large and population-based dataset from patients with COPD regarding S. maltophilia and clinical events. We show, in an unselected, well-characterised, Danish population of COPD outpatients, that a lower respiratory tract sample positive for *S. maltophilia* is associated with a substantial increase in mortality and hospitalisation for exacerbation of COPD. These results were robust to multiple sensitivity analyses and seem biologically plausible. Clinicians should be aware of this pathogen as a possible cause, or at least marker, of clinical decline and events. Randomised controlled trials in which patients with such a positive culture are allocated to a relevant antibiotic therapy or placebo, are strongly encouraged.

### Electronic Supplementary Material

Below is the link to the electronic supplementary material.

Supplementary Material 1

## Data Availability

The data supporting the findings of this study are available from The Danish Health Data Authority following application and approval from The Danish Health Authority. Restrictions apply to the availability of these data, which were used under the license for this study.
