# Supplementary material for: Mortality and exacerbations associated with Stenotrophomonas maltophilia in chronic obstructive pulmonary disease. A regional cohort study of 22,689 outpatients
Source: Respir Res. 2023 Sep 26;24:232. doi: 10.1186/s12931-023-02544-w (PMC10523807; doi:10.1186/s12931-023-02544-w)
Supplement: Supplementary file 1 — Supplementary Material 1 [file 12931_2023_2544_MOESM1_ESM.docx]

**SUPPLEMENTAL APPENDIX 1**

| Comorbidity | ICD-10^†^ codes |
| --- | --- |
| Immunodeficiency | D80-D84, D89 |
| Stroke | I60-I64,  G45, G46 |
| Asthma | J45 |
| Atrial fibrillation | I48 |
| Depression | F32-F34 |
| Diabetes Mellitus | E10-E14 |
| Heart failure | I11.0, I13.0, I13.2, I42, I50, J81 |
| Immunodeficiency | D80-D84, D89 |
| Ischaemic heart disease | I21-I24 |
| Kidney failure | E10.2, E11.2, E12.2, E13.2, E14.2,  I12.0, I13.1, I13.2,  N02-N08, N11, N14, N15.8-N16.5, N16.8, N18, N19, N26, Z99.2 |
| Malignancy | C00-C97, except C44 |
| Peripheral vascular disease | I70.0, I70.2, I70.9, I73.9 I17.0-I17.4, I17.7, I79 |
| ^†^International classification of Diseases 10^th^ revision used for the definition of comorbidities*.* | |

**SUPPLEMENTAL APPENDIX 2**

|  | | | Death | | | Hospitalisation | | |  |
| --- | --- | --- | --- | --- | --- | --- | --- | --- | --- |
|  |  |  | HR | CI | P | HR | CI | P |  |
| *S. maltophilia* | | | 2.6 | 2.2-3.1 | <0.0001 | 2.7 | 2.3-3.1 | <0.0001 |  |
| Sex | | | | | | | | |  |
|  | Female | | ref. | - | - | ref. | - | - |  |
|  | Male | | 1.2 | 1.1-1.2 | <0.0001 | 1.0 | 0.9-1.0 | 0.221 |  |
| Smoking status | | | | | | | | |  |
|  | Non-active smoker | | ref. | - | - | ref. | - | - |  |
|  | Active smoker | | 1.4 | 1.4-1.5 | <0.0001 | 1.4 | 1.3-1.5 | <0.0001 |  |
| Inhaled corticosteroid group† | | | | | | | | |  |
|  | None | | ref. | - | - | ref. | - | | - |
|  | Low | | 1.0 | 0.9-1.1 | 0.808 | 1.5 | 1.3-1.6 | | <0.0001 |
|  | Moderate | | 1.1 | 1.0-1.1 | 0.143 | 1.8 | 1.7-2.0 | | <0.0001 |
|  | High | | 1.2 | 1.1-1.3 | <0.0001 | 1.9 | 1.8-2.2 | | <0.0001 |
| Oral corticosteroid group‡ | | | | | | | | |  |
|  | None | | ref. | - | - | ref. | - | - |  |
|  | Low | | 1.0 | 0.9-1.1 | <0.0001 | 1.7 | 1.5-1.8 | <0.0001 |  |
|  | High | | 1.1 | 1.1-1.2 | <0.0001 | 2.5 | 2.3-2.6 | <0.0001 |  |
| Severe exacerbations | | | | | | | | |  |
|  | 0 | | ref. | - | - | ref. | - | - |  |
|  | 1 | | 1.3 | 1.2-1.4 | <0.0001 | 1.7 | 1.6-1.8 | <0.0001 |  |
|  | ≥2 | | 1.6 | 1.5-1.6 | <0.0001 | 2.6 | 2.4-2.8 | <0.0001 |  |
| FEV1⁎ | | | 0.8 | 0.8-0.8 | <0.0001 | 0.7 | 0.7-0.7 | <0.0001 |  |
| BMI⁑ | | | 0.9 | 0.9-0.9 | <0.0001 | 1.0 | 0.9-1.0 | 0,001 |  |
| Age⁂ | | | 2.1 | 1.9-2.2 | <0.0001 | 1.3 | 1.2-1.3 | <0.0001 |  |
|  | | Risk factors for death or hospitalisation for exacerbation of COPD with acquisition of *S. maltophilia*. HR: hazard ratio, CI: 95% confidence interval, FEV1: forced expired volume in the first second, MRC: Medical Research Council dyspnoea scale, BMI: body mass index. †cumulated dose of budesonide-equivalent ICS 365 days prior to study entry, none: no use, low: ≤400μg, moderate: 400-800μg, high: >800μg, ‡ Oral corticosteroids accumulated dose 365 prior to study entry, none: no use, low dose: ≤ 250 mg prednisolone, high dose: > 250 mg prednisolone, ⁎HR per 10 percentage points increase, ⁑HR per 5 kg/m^2^ increase, ⁂HR per 10 years increase. | | | | | |  |  |
